# Immunoliposomes As a Promising Antiviral Agent against SARS-CoV-2

**DOI:** 10.1134/S1607672923700618

**Published:** 2024-01-07

**Authors:** T. V. Bobik, M. A. Simonova, N. U. Rushkevich, N. N. Kostin, G. A. Skryabin, V. D. Knorre, A. A. Schulga, E. V. Konovalova, G. M. Proshkina, A. G. Gabibov, S. M. Deev

**Affiliations:** 1 Shemyakin–Ovchinnikov Institute of Bioorganic Chemistry, Russian Academy of Sciences, Moscow, Russia; 2 Moscow State University, Moscow, Russia; 3 National Research University Higher School of Economics, Moscow, Russia

**Keywords:** immunoliposomes, virus neutralization, barnase

## Abstract

According to the World Health Organization, as of January 3, 2020 to September 13, 2023, there were approximately 23 million confirmed cases of COVID-19 reported in the Russian Federation, about 400 thousand of which were fatal. Considering the high rate of mutation of the RNA-containing virus genome, which inevitably leads to the emergence of new infectious strains (Eris and Pyrola), the search for medicinal antiviral agents remains an urgent task. Moreover, taking into account the actively mutating receptor-binding domain, this task requires fundamentally new solutions. This study proposes a candidate immunoliposomal drug that targets the S protein of SARS-CoV-2 by the monoclonal neutralizing antibody P4A1 and ensures the penetration of a highly active ribonuclease into the virus-infected cell, which degrades, among cellular RNA, viral RNA too. We demonstrate a more than 40-fold increase in the neutralizing activity of the developed drug compared to the free monoclonal neutralizing antibody.

## INTRODUCTION

In 2020, humanity experienced a pandemic of coronavirus infection SARS-CoV-2. However, we cannot say that the infection has been defeated: new, more aggressive strains of the coronavirus are still emerging in the population, leading not only to infection, but also to lethal outcomes. For example, as of September 2023, the SARS-CoV-2 coronavirus infected more than 770 million people, and the death toll from COVID-19 worldwide exceeded 6.9 million people [1].

To date, a large number of drugs based on neutralizing monoclonal antibodies (nAbs) have been approved for use and continue to undergo clinical trials. Their effect is due to binding to epitopes of the receptor-binding domain of the SARS-CoV-2 S protein, blocking its interaction with the angiotensin-converting enzyme 2 (ACE2) and thus suppressing infection of host cells. An obstacle to the creation of a universal immunodrug is the significant mutagenicity of the RBD domain of the viral S protein. The drugs recommended for the treatment of COVID-19 contain significant doses of recombinant antibodies (400–2400 mg at a single-dose administration) [2], which can provoke adverse events in some cases. Reducing the dose of therapeutic antibodies would thus be justified from both a biosafety and an economic point of view. In this regard, targeted delivery of the drug into a virus-infected cell is of particular importance. One approach to reduce the concentration of a therapeutic antibody is the use of antibody conjugates with a cytotoxic agent. In particular, liposomes loaded with toxins of various natures (organic and inorganic)—immunoliposomes—are used as a cytotoxic agent. This approach was successfully implemented in a number of works [3–5], demonstrating an improvement in the therapeutic index of the drug. It should be noted that the liposomal form of drug delivery has the following advantages: protection of the encapsulated drug from enzymatic degradation and rapid clearance in vivo, reduction of the immunogenicity of protein drugs included in the liposome, and reduction of the overall toxic load from the encapsulated toxin on the body. In addition, the use of immunoliposomes makes it possible to expand the number of potential targets for one type of cytotoxic liposome by simply changing the vector molecule on the liposome surface.

It is known that, due to the interaction of liposomes targeted to the surface viral protein with viral particles and their subsequent fusion, the contents of liposomes are transferred into the viral particle [6]. We assumed that ribonuclease contained in liposomal particles that are targeted to the surface protein of complex RNA-containing viruses can thus be delivered into the contents of the virus and/or infected cell and provide a virus-neutralizing effect due to partial hydrolysis of the RNA contents of the virus and/or infected cell. In this work, we have shown that conjugation of the neutralizing antibody P4A1 [7] with the liposomal particles carrying the ribonuclease increases the neutralizing activity of the P4A1 antibody in the pseudoviral system by more than 40 times.

## MATERIALS AND METHODS

### Preparation of Targeted Liposomal Drugs

Ribonuclease barnase from *Bacillus amyloliquefaciens* was used as a cytotoxic component affecting viral RNA. The loading of barnase into liposomes was based on the electrostatic interaction between the positively charged protein (at neutral pH values) and the negatively charged inner surface of the liposomes. Liposomes were formed from natural phospholipids containing 20% phosphatidylethanolamine. Incubation of a suspension of phospholipids with small hydrophilic and positively charged (at neutral pH) barnase at low ionic strength as a result of squeezing through a polycarbonate filter (mesh, 100 nm) leads to the formation of liposomes with a diameter of approximately 90–100 nm. Modification of the outer surface of liposomes was performed at the amino groups of phosphotidylethanolamine using 2-iminotolan (Trout’s reagent, which allows the incorporation of the SH group at the primary amines of the phospholipid; the final concentration in the reaction mixture was 6 mM) as described in [8]. Recombinant P4A1 antibody or G3 peptide, which was used as a negative control, was conjugated with a 100-fold molar excess of sulfo-EMCS (N-ε-maleimidocaproyl-oxysulfosuccinimide ester, a bilinker allowing incorporation into the protein through the oxysulfosuccinimidemaleimide group). Liposomes containing SH groups, P4A1 antibody, or G3 peptide with the maleimide group on their surface, which were preliminarily purified by gel permeation chromatography (Sephadex G-25 sorbent, Cytiva) from excess of unreacted reagents, were then conjugated to obtain the targeted liposomes P4A1-LB and G3-LB, respectively. The targeting modules P4A1 or peptide G3 that did not react with liposomes were separated from the targeted liposomes by gel permeation chromatography on a column packed with Sepharose CL2B sorbent. Targeted liposomes P4A1-LB and G3-LB leave the column in the exclusion volume, whereas P4A1 and the G3 peptide leave the column in the full volume. The P4A1-L preparation was obtained by conjugating empty liposomes with the P4A1 antibody in a similar manner. By empty liposomes we mean a liposome preparation whose internal aqueous phase does not contain a cytotoxic component.

### Quantitation of P4A1 Antibodies by ELISA

Briefly, 100 μL of a solution of the recombinant receptor-binding domain of the SARS-CoV-2 S protein (RBD) in phosphate-buffered saline (PBS) at a concentration of 1 μg/mL was added to the wells of 96-well MaxiSorp plates (Nunc, Denmark), and the plate was incubated overnight at 2–8°C. After blocking free binding sites with the blocking buffer (PBS, 0.05% Tween-20, and 0.1% BSA), samples of the analyzed drugs and a solution of P4A1 antibody of known concentration in the blocking buffer in dilutions were added, and the plates were incubated for 30 min at 37°C. After 30-min incubation at 37°C and washing, a solution of antibodies to the Fc fragment of human antibodies conjugated with horseradish peroxidase (Sigma Aldrich, United States, cat. no. AP113P) diluted 1 : 10  000 in the blocking buffer was added to the wells, and the plate was incubated for another 30 min. After the end of incubation and washing, 100 μL of TMB substrate solution was added to the wells, and the plate was incubated in the dark for 15 min. The enzymatic reaction was stopped by adding a 10% phosphoric acid solution, and the OD450 values in the wells of the plate were measured using a microplate reader. A curve of the dependence of OD450 values on the concentration of the P4A1 antibody was plotted and used to calculate the concentrations of the P4A1 antibody in the liposomal samples.

### Determination of Neutralizing Activity in a Pseudoviral System

Neutralizing activity was assessed using pseudotyped lentiviruses carrying the SARS-CoV-2 S protein [9] as described in [10] using P4A1 antibody solutions of known concentration as a control. Serial samples of the analyzed liposomal drugs were prepared by diluting the studied drugs in DMEM with 10% FBS. The curves of the dependence of luminescent signal values on drug dilutions were constructed using the GraphPadPrism 8 software, and the concentration of the substances that ensured neutralization of the pseudovirus IC_40_ was calculated.

### Determination of the Cytotoxic Effect of Liposomes In Vitro

Cytotoxic effect of liposomal drugs on HEK293T-ACE2 cell was determined using the standard MTT test [11]. Cells were seeded in a 96-well plate at a density of 1.5 × 10^3^ cells/well and cultured overnight. The growth medium was replaced with a fresh one containing liposomes at different concentrations (0, 0.005, and 0.05 nM), and the cells were incubated for 72 h at 37°C. After the end of incubation, the cells were washed with phosphate-buffered saline and incubated in a fresh medium containing 0.5 mg/mL MTT for 1 h. The formed formazan crystals were dissolved in dimethyl sulfoxide. The absorbance in the wells was measured using a Tecan Infinite plate reader at 570 nm. Relative cell viability was calculated as the ratio of the average optical density in the wells with treated cells to the average optical density in the wells with untreated (control) cells and expressed as a percentage.

### Data Processing

The figures (unless otherwise indicated) present data from at least three independent experiments. Data were processed using the GraphPadPrism8 software (GraphPad Software, United States).

## RESULTS AND DISCUSSION

**Fig. 1.**
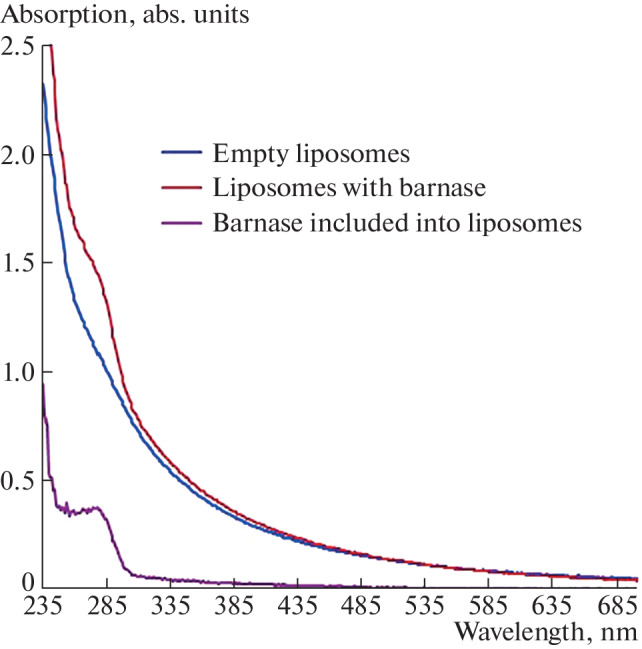
Absorption spectrum of liposomes. Red and blue curves are the absorption spectra of liposomes loaded with barnase and empty liposomes, respectively. The purple curve is the absorption spectrum of barnase included into liposomes. This spectrum was obtained by subtracting the absorption spectrum of empty liposomes from the absorption spectrum of proteoliposomes.

The loading of barnase into liposomes was assessed spectrophotometrically. To do this, the spectrum of empty liposomes was subtracted from the absorption spectrum of proteoliposomes (Fig. 1). Protein concentration was calculated using the molar extinction coefficient ε_280_ = 26  930 M^–1^ cm^–1^. The concentration of barnase in liposomes was 13.3 μM. The concentration of the barnase-loaded liposomes was assessed spectrophotometrically by comparing the spectrum of empty liposomes and proteoliposomes.

As can be seen in Fig. 1, the spectrum of proteoliposomes coincides with the spectrum of empty liposomes obtained by 17-step squeezing a suspension of phospholipids (1.1 mg/mL) through a filter with a pore diameter of 100 nm. Previously, using the hydrophilic membrane-impermeable dye copper phthalocyanine-3,4',4',4''-tetrasulfonate, we showed that 1 mg/mL of a suspension of lipid vesicles corresponds to 1.2 nM [8]. Since the spectrum of the barnase-loaded liposomes coincides with the spectrum of empty liposomes with a concentration of 1.1 mg/mL, the concentration of the protein-loaded liposomes is 1.3 nM. To selectively eliminate pseudoviral particles containing the S protein gene on their surface, the outer surface of the liposomes was covalently modified with the recombinant neutralizing antibody P4A1, specific to the S protein of the SARS-COV-2 virus (P4A1-LB).

The P4A1 antibody was obtained by screening B cells of an individual who had undergone COVID-19 [7]. This antibody specifically and effectively (KD = 1.02 × 10^–10^ M) binds the receptor-binding domain (RBD) of SARS-CoV-2 S protein, blocking its interaction with ACE2. To assess the specificity of the P4A1-LB drug, the G3-LB drug was prepared by covalently modifying the outer surface of barnase-containing liposomes with the G3 peptide. Peptide G3 is a protein of non-immunoglobulin nature, capable of highly specific (KD = 0.9 × 10^–9^ M) interaction with human epidermal growth factor receptor II [12]. To assess the contribution of barnase to the neutralizing activity of the P4A1-LB drug, we obtained the P4A1-L drug by covalently modifying the outer surface of empty liposomes with the P4A1 antibody. The neutralizing activity of the obtained drugs, as well as the intact P4A1 antibody, was assessed in a pseudoviral system using SARS-CoV-2 S protein-pseudotyped lentiviruses encoding firefly luciferase (Luc) [10].

**Fig. 2.**
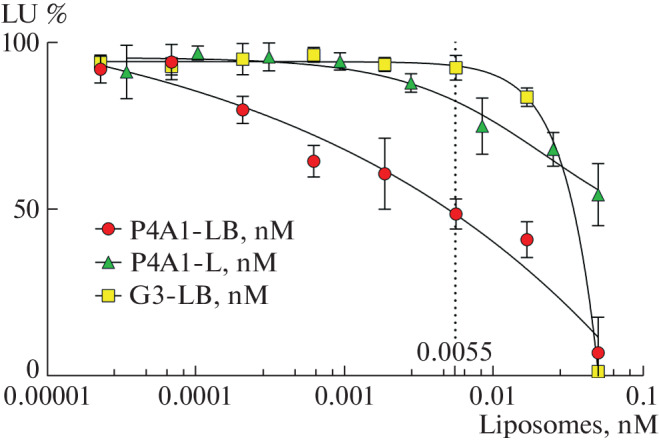
Dependence of the intensity of luminescence of HEK293T-ACE2 cells expressing the human ACE2 receptor on the surface on the concentration of liposomal drugs in a pseudovirus system.

All three studied drugs exhibited neutralizing activity, which was apparently determined by different natures (Fig. 2). In the case of the G3-LB drug, which has the G3 peptide as a guiding molecule and is unable to bind to the SARS-CoV-2 S protein, neutralizing activity is manifested at a liposome concentration higher than 0.0055 nM. This effect is possibly realized due to the nonspecific binding of liposomal particles to the surface of pseudoviruses and/or cells and subsequent fusion with them. Barnase, which is released after fusion, exhibits RNase activity against the viral genome in pseudoviruses and/or cytoplasmic RNA of infected cells, which leads to a decrease in the synthesis of the luciferase marker protein. To rule out a possible explanation for the observed decrease in luminescence by cell death due to the cytotoxic effect of nonspecific internalization of barnase-containing liposomes, we studied changes in the viability of HEK293T-ACE2 cells treated and not treated with G3-LB, P4A1-LB, and P4A1-L preparations using standard MTT test [11]. The data obtained revealed no statistical difference in the viability between the treated and untreated HEK293T-ACE2 cells for all studied drugs, which indicates the absence of a significant cytotoxic effect of the studied liposomal drugs in the concentration range of 0–0.05 nM.

**Fig. 3.**
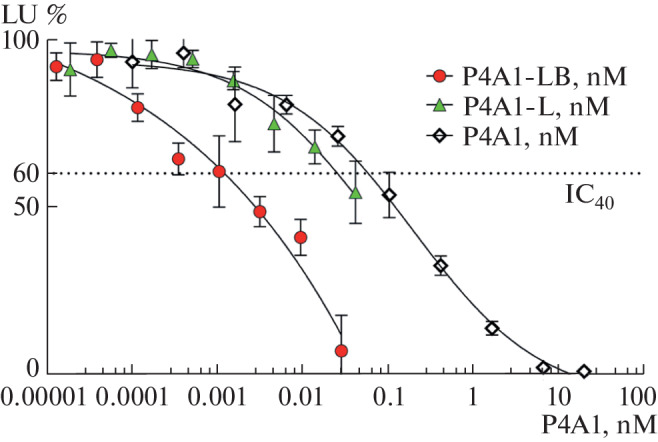
Dependence of the intensity of luminescence of HEK293T-ACE2 cells expressing the human ACE2 receptor on the surface on the concentration of antibody P4A1 in liposomal drugs and buffer solution in a pseudovirus system.

In the case of the P4A1-L drug, which does not contain barnase, neutralizing activity is due to the concentration-dependent blockade by the P4A1 antibody of the SARS-CoV-2 S protein on the surface of pseudoviruses, which, in turn, prevents interaction with the ACE2 receptor and further penetration of pseudoviruses into cells. In the case of the P4A1-LB drug, we can assume that the neutralizing activity is due to the synergistic effect caused by the concentration-dependent blockade of the penetration of pseudoviruses, as in the case of the P4A1-L drug, and the RNase activity of barnase, as in the case of the G3-LB drug. To correctly compare the neutralizing activity of the resulting liposomal preparations P4A1-LB and P4A1-L, the concentration of the conjugated antibody P4A1 in the preparations was determined by ELISA. Since for the P4A1-LB drug, luminescence intensity values for P4A1 concentrations above 0.0028 nM, corresponding to liposome concentrations above 0.0055 nM, may be underestimated due to the influence of nonspecific internalization of barnase, the comparison of neutralizing activity was performed by determining the 40% inhibitory concentration (IC_40_). According to the data obtained (Fig. 3), the neutralizing activity of the liposomal preparations P4A1-L and P4A1-LB (IC_40_ 17 ± 9 and 1.2 ± 0.9 pM, respectively) is higher than that of the free P4A1 antibody (IC_40_ 55 ± 6 pM). These data suggest that liposomal particles significantly contribute to neutralizing the interaction of the pseudovirus S protein with the ACE2 receptor. This effect can be explained by several mechanisms.

It is possible that liposomes effectively shield the interaction of free S-protein molecules with the ACE2 receptor, preventing cell infection. The effect of “extraction” of S-protein molecules from the surface of pseudoviruses by liposomal particles is also possible [13]. The significant increase in the neutralizing activity of P4A1-LB (IC_40_ 0.12 ± 0.09 pM) compared to that of P4A1-L (IC_40_ 1.7 ± 0.9 pM) is due to the manifestation of RNase activity with respect to the viral genome of pseudoviruses and/or RNA molecules of infected cells due to the release of barnase from liposomal particles.

Thus, as a result of this work, immunoliposomes targeted to the SARS-COV-2 S protein by the recombinant neutralizing antibody P4A1, containing (P4A1-LB) and not containing (P4A1-L) RNase barnase, were obtained. It is shown that liposomal preparations P4A1-LB and P4A1-L have neutralizing activity higher than the activity of the free neutralizing antibody P4A1. The immunoliposome preparation P4A1-LB has a neutralizing activity more than 40 times higher than the activity of the neutralizing antibody P4A1. The approach proposed in this article is demonstrated using liposomes targeted to a specific virus as an example. However, this methodology, which includes the use of the P4A1 antibody as a targeting molecule, cannot be considered as a universal means for creating anti-COVID drugs due to mutagenesis, which usually leads to changes in the parameters of interaction of already known binding and neutralizing antibodies (including P4A1) with viral proteins. Therefore, to create drugs in this and other cases, studies of the specificity of the interaction between the targeted liposome and the target on the surface of the virus will be required.
